# Whole Grain Consumption for the Prevention and Treatment of Breast Cancer

**DOI:** 10.3390/nu11081769

**Published:** 2019-08-01

**Authors:** Mingsi Xie, Jie Liu, Rong Tsao, Ziyuan Wang, Baoguo Sun, Jing Wang

**Affiliations:** 1 State Key Laboratory of Food Nutrition and Safety, Tianjin University of Science & Technology; School of Food Engineering and Biotechnology, Tianjin University of Science & Technology, Tianjin 300457, China; 2 China-Canada Joint Lab of Food Nutrition and Health (Beijing), Beijing Technology & Business University (BTBU), Beijing 100048, China; 3 Guelph Research and Development Centre, Agriculture and Agri-Food Canada, Guelph, N1G 5C9, Canada

**Keywords:** breast cancer, whole grains, bioactive compounds

## Abstract

Breast cancer is one of the most common and malignant cancers among females worldwide. Several epidemiological studies have indicated the inverse correlation between the intake of whole grains and the incidence of breast cancer. Whole grains are the most fundamental and important food source of bioactive phytochemicals, which have well-defined roles in the management of each stage of breast carcinogenesis. To better understand the value of whole grains in future prevention and treatment of breast cancer, the effects and possible mechanisms of six different whole grain cereals, which are the most commonly consumed throughout the world, are introduced in the current review. Moreover, the bioactive compounds extracted from whole grains are adequately formulated and the underlying mechanism of action is illustrated. In addition, the present limitations and future perspective of whole grain consumption for breast cancer are also concluded. The objective of this review is to promote the development of nutraceutical and functional food from whole grains and its application for reducing the risk of breast cancer.

## 1. Introduction

Breast cancer is the most frequently diagnosed cancer among women worldwide and has become an increasing global health issue over the past few decades. The World Cancer Research Fund (WCRF) states that dietary habits have a critically important role in the prevention and causation of cancer [1]. Epidemiological studies have showed that breast cancer is more prevalent in Europe and North America than in Asian countries [2,3]. One of the most important reasons for this may be ascribed to different dietary patterns. There are many evidences that support the inverse relation between whole grain intake and breast cancer risks [3,4,5,6], indicating that whole grains might hold nutraceutical characteristics against breast cancer. As a result, many efforts have been made in the identification and characterization of phytochemicals from whole grains with potential chemo-preventive properties. However, the relationship between whole grains and breast cancer remains not fully understood. Besides, previous research on this topic does not link closely with the current development in cancer prevention and management, such as synergistic application with adjuvant therapy. Therefore, a systematic review over the current research status of preventive effects of whole grains (and its products) against breast cancer is urgently needed.

In view of this, the main objective of this review is to summarize and provide an overview of the chemo-preventive effects of whole grain products and their bioactive compounds against breast cancer and promote their applications for the prevention and management of breast cancer. Considering the fundamental status of whole grains in citizens’ livelihood, the development of whole-grain functional foods is especially important and beneficial for human health. The findings might also be suggestive for guiding policy initiatives and nutritional suggestions for cancer prevention. 

## 2. Breast Cancer

### 2.1. General Aspect of Breast Cancer

Cancer remains a major cause of mortality and morbidity around the world [7]. Breast cancer is one of the most frequently diagnosed carcinoma in women [1]. According to data of GLOBOCAN 2018, there were 2.1 million newly diagnosed breast cancer cases in 2018, representing a quarter of all new cancer cases in women worldwide [8]. In the United States, there are 268,600 newly diagnosed cases, representing 30% of all new cases in women, and 41,400 deaths, ranking second in the U.S. in 2019 [2,9]. In the meantime, breast cancer death risk ranks the second in all-age U.S. women and first in women between the ages of 20–59 [2]. Moreover, breast cancer is the most frequently diagnosed cancer and the sixth leading cause of death in Chinese women [10,11]. In 2015, there were approximately 270,000 new cases of breast cancer, accounting for 15% of newly diagnosed cancers in women, and 69,500 deaths, making it the leading cause of death in Chinese women younger than the age of 45 [10]. Although, the mortality of breast cancer in North America and Europe has dropped due to advances in early detection and systemic endocrine and targeted therapies. However, the breast cancer toll is still increasing in China [12]. 

Breast cancer is a complex disease featuring distinct histological, clinical, and molecular phenotypes. A lot of aspects can affect breast cancer development, including intrinsic factors, e.g. family history and genetic mutation, as well as extrinsic factors, e.g. environment, radiation, living styles, and reproduction condition [1,13,14,15,16]. With technology advances in the last decade, researchers have revealed that breast cancer is not just one single disease, but a heterogeneous group of imbalances, and therefore, the responses to different therapeutic treatments vary significantly [17,18]. According to the molecular receptor status (estrogen receptor (ER), progesterone receptor (PR), and human epidermal growth factor receptor-2 (HER-2)) and the gene expression detected by multiple microarray platforms, breast cancer is categorized into various subtypes: luminal A (ER/PR-positive/HER-2-negative), luminal B (ER-positive, PR-positive or negative, and HER-2-positive), basal-like (ER/PR/HER-2-negative), normal breast-like (ER/PR-positive or negative and HER-2-negative), HER-2-enriched, and claudin-low (ER/PR-negative, HER-2-positive, and low expression of claudin) (Table 1) [19,20,21]. Luminal breast cancer (luminal A and luminal B) are featured with positive expression of estrogen (ER) and/or progesterone (PR). Compared with luminal A, luminal B is more aggressive and worse in prognosis. Both subtypes show high expression of luminal-related genes (e.g. ESR1, KRT8) and of ER transcription factors (e.g. GATA3, FOXA1). HER-2-enriched subtype is characterized by positive expression of human epidermal growth factor receptor 2 (HER-2) as well as negative expression of ER. HER-2-enriched breast cancer cells have high expression of genes including HER-2, GRB7, and have higher migration ability compared with luminal cells. Lacking ER, PR, and HER-2, triple-negative breast cancer cells are the most heterogeneous among all intrinsic subtypes of breast cancer. Basal-like subtype is the major one of the basal tumor subtypes for its high expression in basal gene markers such as KRT5, LAMC2, FABP7, etc. Normal basal-like and claudin-low subtype represent the mesenchymal subtype cluster. These subtypes are highly expressed in gene markers featured in highly invasive and aggressive breast cancer phenotypes such as VIM, MMP2/14, COL3A1, CD24(-), and CD44(+).Classification based on gene expression profile is still developing [22,23], which makes characterization of breast cancer even more complex. The distinct subtypes of breast tumor respond differently to treatments, making breast cancer extremely challenging. 

Breast cancer mortality has declined significantly in Western countries in the past few decades due to early diagnosis and effective adjuvant therapies [15,24,25]. Currently, for early stage breast cancer, treatment strategies include both local (surgery and radiotherapy) and systemic therapies that combine endocrine, targeted, and chemotherapies. Conventional cytotoxic therapies, including chemotherapy and radiation therapy, are the most common choices for cancer management. However, many women still suffer from relapse and tumor metastasis. The efficacy of conventional chemotherapy and radiotherapy is far from satisfactory due to drug/radiotherapy resistance and side effects. Developments of novel anticancer agents and alternative approaches with high selectivity and therapeutic indices and relatively low toxicity have become a new direction for cancer chemotherapy research and development [26]. There is an urgent call for novel and effective prevention and treatment strategies for breast cancer. 

### 2.2. Molecular Mechanism of Breast Cancer Treatment

#### 2.2.1. Proliferation Inhibition

One major feature of cancer is a dysregulated and aggressive proliferation of the tumor cells. In normal and healthy cells, proliferation is precisely regulated through a balance between the growth and antigrowth signals. However, cancer cells develop the ability to grow uncontrollably, which generate their own growth signals and become insensitive to antigrowth signals. Apoptosis is a programmed cell death process that is continually occurring in normal cells [27]. The caspases and the members of the Bcl-2 family of proteins were involved in modulating cells’ apoptosis [28]. Studies have proven that the mitogen-activated protein kinase (MAPK), the phosphoinositide 3-kinase (PI3K)/Akt, and the epidermal growth factor receptor (EGFR) signal transduction cascades function abnormally in most tumor cases and therefore, they became key targets for targeted therapy-mediated cell death [29]. The PI3K-AKT-mTOR pathway is a central knot in the transduction of extracellular and intracellular growth signals. Inhibition of PI3K pathway signaling reduced cancer cell growth and survival [30]. 

#### 2.2.2. Immune System Modulation

There is clear evidence that the immune system and inflammation play a critical role in the process of carcinogenesis. An inflammatory microenvironment is an essential component of all tumors [31]. Inflammatory responses are involved in every stage of cancer development and progression, such as initiation, promotion, malignant transformation, invasion, and metastasis [32]. The immune system can eliminate premalignant and transformed cancer cells. However, cancer cells can bypass the immune system through the development of resistant or immunogenic clones. Various immune cells are frequently found accumulated in tumors relative to the surrounding tissue. These immune cells infiltrate tumors and communicate with tumor cells [33]. The important link between inflammation and carcinogenesis is the pro-inflammatory transcription factors. Moreover, the inflammatory mediators such as pro-inflammatory cytokines also stimulate the survival and proliferation of premalignant cells and activate oncogenic transcription factors [34]. Blocking pro-inflammatory cytokines or endotoxin-mediated kinases and transcription factors involved in cancer progression could inhibit inflammation and cancer recurrence.

#### 2.2.3. Targeting Metastasis and Breast Cancer Stem Cells

Emerging studies have revealed that breast cancer stem cells’ (BCSCs) subpopulation and their epithelial-mesenchymal transition (EMT) behavior are major reasons for resistance and migratory potential [35,36,37]. Conventional chemotherapy would increase the percentage of CD44^high^/CD24^low^ tumor cells in breast cancer patients, which acts as a breast cancer stem cell marker [38]. Mammary cells developed into BCSCs by EMT [36,39,40], a process by which epithelial cells transit into mesenchymal phenotype, allowing them to be free from the primary tumor site and metastasize at distant sites [39]. EMT signaling is involved in the development and maintenance of BCSCs. BCSCs and EMT are involved in a crosslinked signaling network, including TGF-β, Wnt, Notch, NF-κB, and ERK/MAPK pathways [41]. Apart from the intrinsic signal pathway, BCSCs are also regulated by the tumor microenvironment [35], which contains inflammatory cells, BCSCs, fibroblasts, and cytokines. Several inflammatory cytokines, including IL-6 and IL-8, have been reported to regulate breast cancer stem cell self-renewal through the cytokine loop [42]. Developing strategies to interfere with cytokines and their receptors are in progress to target breast cancer stem cells.

## 3. Anti-Breast-Cancer Efficacy of Whole Grains

### 3.1. General Health Benefits of Whole Grains

Many foods are considered as key elements for cancer prevention, and phytochemicals in whole grains are especially important [1]. Whole grains are the edible seeds of the grass family plants, which are composed of the embryo (or germ), endosperm, starch granules, as well as the outside bran (fiber). Grain plants are highly adaptable to the environment and thus are widely cultivated in different climatic and geographical conditions. Wheat, rice, millet, rye, barley, sorghum, oats, and maize are the most fundamental and important source of food and energy around the world. Grains are rich in carbohydrates, proteins, minerals, and vitamins. 

**Figure 1:**
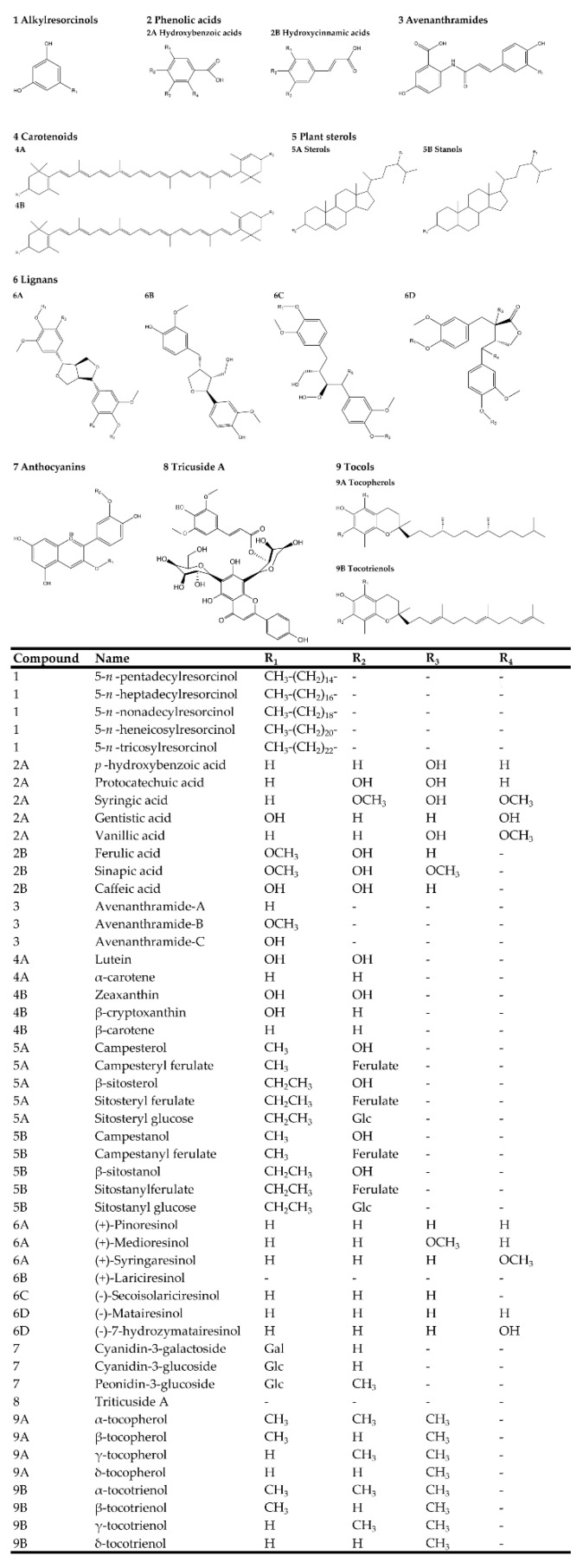
Major bioactive phytochemicals derived from whole grains.

Consumption of whole grains has been proven to decrease the risk of obesity, diabetes, and cardiovascular diseases [43,44,45,46,47,48,49,50,51,52,53,54]. Over the last few decades, accumulating evidence has shown that high intake of whole grain products prevents cancer occurrence [53,55,56,57,58]. These health benefits of whole grains could be attributed to the abundant phytochemicals in different cereal species [59]. The major bioactive phytochemicals found in grains are listed in Figure 1. There has been a huge amount of literature describing the benefits of whole grains including antioxidant, anticancer, anti-inflammatory, as well as illness risk reduction activity [60,61,62,63]. These health benefits are briefly reviewed in Table 2. 

### 3.2. Epidemiological and Clinical Studies of Whole Grain Consumption and Breast Cancer 

Clinical epidemiological studies of whole grain consumption and breast cancer have been summarized in Table 3. A recent meta-analysis suggested that a healthy dietary pattern could lower breast cancer risk, especially in postmenopausal, hormone receptor–negative women [6]. Epidemiological studies also suggest that the consumption of whole grains is related to a reduced risk of breast cancer [81]. A recent meta-analysis including four cohort and seven case-control studies suggested that high whole grains intake might have an inverse association with breast cancer risk (RR (relative risks) = 0.84; 95%CI = 0.74–0.96; *P* = 0.009; *I^2^* = 63.8%). Further stratified analysis indicated that the inverse association was only observed in case-control studies (RR = 0.69; 95%CI = 0.56–0.87; *P* = 0.001; *I^2^* = 58.2%), but not in cohort studies (RR = 0.96; 95%CI = 0.82–1.14; *P* = 0.69; *I^2^* = 66.7%) [5]. Two other reviews and meta-analyses of prospective studies correlate dietary fiber intake and reduced breast cancer risk [55]. Data from a more recent cohort analysis of American females suggested an inverse relation between whole grains (or dietary fiber intakes) and breast cancer risks in both adolescent population and adults before menopause [81,82]. Further stratified analysis indicated that higher adult intake of whole grains was associated with lower premenopausal breast cancer risks (RR = 0.82; 95%CI = 0.70–0.97), and that an inverse relation between higher adolescent and early-adulthood intake of whole grains and breast cancer risks (RR = 0.74; 95%CI = 0.56–0.99) existed. Adulthood consumption of brown rice was linked with lower overall risks of breast cancer (2 servings/week: RR = 0.94; 95%CI = 0.89–0.99). A case-controlled study from Greece also suggested that whole grains consumption more than 7 times/week was consistently associated with reduced risk of breast cancer (OR (odds ratios) = 0.49; 95% CI = 0.29-0.82) for women between 44–68 years old [6]. Another case-control study from Iran suggested that the consumption of resistant starch (RS)-containing food, such as whole grain bread, could reduce breast cancer risk (OR = 0.61; 95%CI = 0.37–0.99) for women aged from 25 to 65 years [83]. A 2017 cohort study showed that the consumption of whole grain food may protect against breast cancer risk with a 47% reduction rate (HR (hazard ratios) = 0.53; 95%C I = 0.33–0.86), while consumption of whole grain food had no clear association with other adiposity-related cancers [56]. Dietary intervention with whole grains and related products could be a pragmatic approach for breast cancer prevention and management. Their inherent safety makes whole grain foods an appealing choice for widespread, long-term use in diverse populations.

Although results of most epidemiological studies support an inverse relationship between whole grain (or dietary fiber) consumption and breast cancer risks, more evidences are needed to draw firm conclusions. Controversies still exist. A review of five cohort studies found that there is no solid association between whole grain consumption and breast cancer risks, and there is no statistically significant relationship between whole grain fiber consumption and breast cancer risk in any of the 11 cohort studies conducted in Europe and North America [57]. Among all studies above, four studies announced a significant decrease in cancer risk, yet one study reported a non-significant increase in cancer risk with a heavy intake of whole grains. A possible explanation for this inconsistency in results might be varied study designs and a lack of risk factor adjustments. Moreover, the assessment standard of whole grains (or grain fibers) intake was changed between the different studies [57]. The inability to measure whole-grain intake might lead to diverse, even opposite results from existing evidence. Therefore, more large-scale cohort studies with more standardized whole grain intake assessment methods are needed to confirm the relationship between grains intake and breast cancer risks in the future.

### 3.3. Whole Grain Phytochemicals and Anti-Breast-Cancer Property

**Figure 2:**
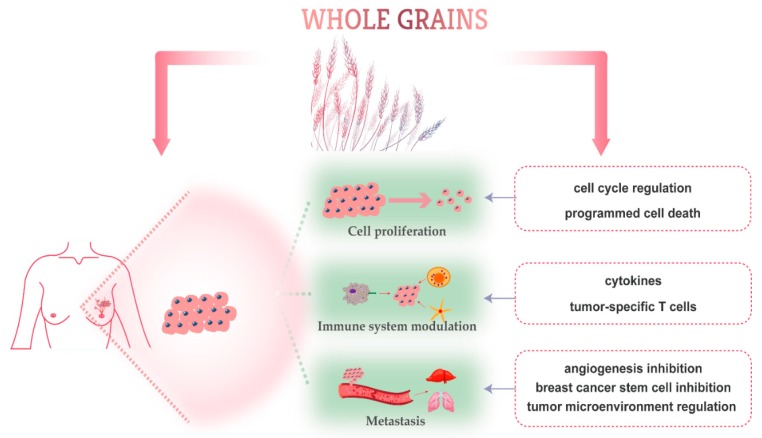
Schematic overview of molecular-targeted mechanisms of phytochemicals derived from whole grains against breast cancer.

Phytochemicals are non-essential nutrient bioactive components found in plant foods. Whole grains are a rich source of phytochemicals such as phenolic acids, carotenoids, alkylresorcinols (ARs), phytosterols, lignans, anthocyanins, vitamin E member, and polysaccharides (Figure 1) [85]. The anti-cancer activities and potential health benefits can be attributed to the abundant bioactive phytochemicals in whole grains [57]. Whole grains’ dietary fiber function directly in the small intestine and colon, therefore their effects on colorectal carcinogenesis are most intensely studied [52,58,86,87,88]. Unlike dietary fiber, the anticancer activity of some phytochemicals is still needed for future comprehensive investigations [89]. Several experimental studies have shown that bioactive components of whole grains exert anti-breast cancer activity through inhibiting proliferation, modulating immune system, and inhibiting metastasis of breast tumor cells [90]. Below, we will overview some of the most common whole grains and discuss their role in breast cancer prevention (Table 4) and the major breast cancer molecular mechanisms targeted by whole grain-derived phytochemicals (Figure 2).

#### 3.3.1. Wheat

Wheat is the most cultivated crop in the world [104]. Wheat grains and their processed products are staple foods and are one of the most important dietary energy sources. Components of germinated wheat flour have been shown to have an inhibitory effect against the human breast cancer cell line MCF-7(ER+) and MDA-MB-231 (TNBC, triple negative) by up-regulating apoptosis of both cell lines [91]. ARs, or 1,3-dihydroxy-5-n-alkylbenzenes, are one major group of phenolic lipids found in whole grain wheat, barley, and rye. ARs can be absorbed and detected in plasma. Thus, ARs can be used as biomarkers for whole grain wheat/rye consumption [105,106,107]. A wide range of bioactive properties of ARs have been reported including antioxidative and anti-carcinoma activities, suppressing adipocyte lipolysis, obesity reduction, and increasing glucose tolerance and insulin sensitivity [64,65,105,108,109,110,111,112,113]. A recent paper reviewed the relation between dietary alkylresorcinols and cancer prevention, concluding that alkylresorcinols are likely to be useful in impeding cancer progression [114]. A series of ARs (C17:0–C25:0) isolated from wheat bran oil showed growth inhibition potential against human colon cancer cell HCT-116 and HT-29 [64], indicating that wheat bran ARs do have an anti-carcinoma ability. There are a few preliminary in vitro studies proving that ARs could inhibit the growth of human breast cancer cell. Five ARs isolated from *Homalomena wendlandii Schott(Areacae)* inhibited the growth of the breast cancer adenocarcinoma cell line MCF-7 with IC_50_ values ranging between 8.24–42.17μM [115]. Currently, there have been no direct studies conducted to evaluate the effect of grain-originated ARs on breast cancer, yet the effect is predictable. Besides ARs, triticuside A, a flavone C-glycoside from wheat bran, strongly suppressed the proliferation of human breast cancer cells (MCF-7 and MDA-MB-231) via the mitochondrial apoptosis pathway and the Akt/mTOR signaling pathway [95]. 

#### 3.3.2. Rice

Rice is a major part of the global food supply, serving as a staple for over 50% of the world’s population. In Asia, nearly half of the grains consumed annually is rice. Unlike white rice with the husk, bran, and germ removed, whole grain rice like red rice and black rice contain higher contents of bioactive phytochemicals, such as phenolics, oryzanol, tocotrienols, and tocopherols that possess various beneficial health activities. Black rice anthocyanins significantly suppressed breast cancer metastasis in several studies [40,71,103]. In a recent study, black rice anthocyanins were shown to inhibit HER-2 breast cancer cell metastasis by suppressing cancer cell growth, migration, and epithelial-mesenchymal transition via the cSrc/FAK/p130^Cas^ signaling pathway [40]. Red mold rice, fermented by *Monascus purpureus* NTU 803, is a traditional food and folk medicine in East Asia. Extracts of red mold rice exhibited direct cytotoxic and proapoptotic effects on breast cancer cell MCF-7 through activation of Caspase-9 and Caspase-3 of the mitochondria-dependent pathway in a time-dependent manner [116]. 

A recent study indicated that black rice anthocyanins could suppress HER-2-positive breast cancer cells invasion by targeting the RAS/RAF/MAPK pathway, a pivotal signaling pathway in breast cancer development [71]. Black rice anthocyanins inhibited HER-2+ MDA-MB-453 human breast cancer cell migration and invasion, suppressed the activation of RAF, MEK, and JNK, and downregulated the secretion of MMP-2 and MMP-9. In another study, proanthocyanin, γ-oryzanol, and γ-tocotrienol extracted from red rice fractions were found to have anti-invasion activity against HT1080 and MDA-MB-231 cancer cells by decreasing the expression and activity of matrix metalloproteinase-2 and -9 (MMP-2 and -9) [98]. Black rice anthocyanins also exerted anti-metastasis potential against human breast cancer cells by reducing transplanted tumor growth and inhibiting pulmonary metastasis of breast cancer xenografts and decreasing urokinase-type plasminogen activator (u-PA) activity [103]. Recent studies also suggest that bioactive peptides of food origin have been shown to play important roles in the prevention and treatment of cancer and cardiovascular and infective diseases [117]. Many peptides extracted from whole grains exhibit anticancer potential, however most related studies focus on efficacy over gastrointestinal cancer [118]. In one study, a pentapeptide with a sequence of Glu-Gln-Arg-Pro-Arg, isolated from rice bran, showed antiproliferative characteristics against both MCF-7 and MDA-MB-231 cells [99].

#### 3.3.3. Sorghum

Sorghum is the fifth most economic cereal crop in the world, and the health benefits of its unique phytochemicals have been under intense investigation in recent years [68,119,120]. Various human health benefits associated with sorghum can be attributed to its abundant secondary metabolites such as anthocyanins, phenols, tannins, phytosterols, and policosanols [121]. Anthocyanins extracted from red sorghum bran showed an anti-proliferative effect against MCF-7 cancer cells by inducing apoptosis, as revealed in the formation of apoptosis body and DNA fragmentations [101]. A similar result was found in another study where red-sorghum-bran 3-deoxyanthocyanin inhibited MCF-7 cells viability by upregulating the p53 expression and down regulating the Bcl-2 expression [102]. Sorghum extract suppressed the growth of MDA-MB-231 and MCF-7 cells through inducing the G1 cell cycle phase arrest, down-regulating the STAT5b/IGF-1R and STAT3/VEGF pathways, and inhibiting metastasis in BALB/C nude mice bearing breast cancer xenografts [100]. Results showed that tumor growth was suppressed by sorghum extracts through modulating JAK/STAT pathways and downregulating the expression of angiogenic factors like VEGF, VEGF-R2, and cell cycle regulators like cyclin D, cyclin E, and p-Rb. The same study further showed that sorghum extracts exhibited significantly higher anticancer bioactivity than other grains like wheat, millet, and panicum, indicating that sorghum might serve as an effective and inexpensive edible supplement in cancer management [100]. Sorghum 3-deoxyanthocyanidins were proven to be more cytotoxic to cancer cells than anthocyanidins from other foods [122]. Apart from 3-deoxyanthocyanidins, sorghum tannins also have anticancer potential. In one study, sorghum bran extract, which was rich in tannins, inhibited aromatase activity, a key enzyme in estrogen synthesis and an important target for breast cancer chemotherapy [123]. Further studies are needed for the anticancer effect of sorghum tannins.

#### 3.3.4. Oat

Oats are an outstanding source of soluble dietary fibers such as β-glucans and health-beneficial phytochemicals. Oat β-glucans have been intensely studied against multiple cancer cells [124,125] and in animal models [79]. Oral administration of β-glucans showed an immunomodulatory effect by stimulating peripheral blood monocytes proliferation in advanced breast cancer patients [126]. However, the relation between oat β-glucan consumption and breast cancer still needs to be further explored. Avenacosides, a unique group of steroidal saponins from oats, have drawn growing research attention for their chemopreventive potential against human colon cancer cells [127]. Among all phytochemicals of oats, avenanthramides are uniquely found in oats. Avenanthramides are found to possess a wide range of health benefits [67]. Avenanthramide-C was shown to reduce the viability of MDA-MB-231 breast cancer cells through the induction of sub G1 cell cycle arrest and apoptosis, causing DNA fragmentation and activation of Caspases [94]. Dihydroavenanthramide D, a synthetic analog of avenanthramides, suppressed breast cancer growth by inhibiting MCF-7 cancer cell invasion through the MAPK/NF-κB and MAPK/AP-1 pathway [93].

#### 3.3.5. Other Grain Species

Consumption of other cereals and phytochemicals have also been found to have positive impacts on breast cancer risk reduction and anti-proliferative activities. Barley consumption, for example, has been shown to improve health [45]. In one study, barley exhibited anti-tumor activities in both the rat mammary tumor model and MCF-7 cell line through the induction of cell cycle arrest, pro-apoptosis, and the antiproliferation mechanism [92]. Young barley significantly increased caspase-3 and reduced Ki67 expression in rat tumors [92]. Millet is also an important small-seed grain crop, which is widely cultivated in China and East Asia, but its health benefits are greatly underestimated. Phenolic extract of foxtail millet was found to strongly inhibit the proliferation of MDA-MB-231 breast cancer cells [61]. However, more research needs to be conducted on the specific anti-breast cancer components and their role in chemoprevention.

#### 3.3.6. Synergistic Effects of Whole Grain Phytochemicals and Anti-Breast-Cancer Therapy Agents

Current cancer therapeutic strategies often do not receive good and stable efficacy, due to toxicity, resistance, and tumor metastasis. Chemotherapy is one of the most frequently used treatments in cancer management. However, it is often followed by adverse effects and resistance, which is the major causes for disease recurrence. Some whole grain products and related phytochemicals have shown synergistic effects with chemotherapy, either by sensitizing the tumor cells or by reducing their side effects. Wheat grass juice taken by breast cancer patients under combined chemotherapy (5-fluorouracil, doxorubicin, and cyclophosphamide) was found to have lowered levels of toxicity and reduced chemotherapies dosage, without decreasing their efficacy [128]. Two rice bran components, δ-tocotrienol and ferulic acid, synergistically inhibited the proliferation of human breast cancer cell MCF-7, while ferulic acid alone did not show any anti-proliferative effect [96]. MGN-3/Biobran, arabinoxylan acquired from rice bran, increased the susceptibility of MCF-7 cells and murine metastatic breast cancer cell 4T-1 to paclitaxel by more than 100-fold, resulting in increased DNA damage and apoptosis [97]. MGN-3/Biobran was also reported to increase the susceptibility of human breast cancer cells MCF-7 and HCC70 to daunorubicin by enhancing drug accumulation in cancer cells [129].

## 4. Discussion 

In this review, we described literature investigations on cereal bioactivity against breast cancer. We have also overviewed molecular mechanisms of cereal phytochemicals, offering new options for adjuvant therapy development. Whole grain cereals may reduce breast cancer risk via multiple ways. Firstly, whole grains could influence the obesity situation by promoting satiation and satiety, reducing caloric intake, and controlling body adiposity due to its high fiber content, which can lower energy density [130,131]. Abundant fiber content in whole grains could significantly control insulin resistance and insulin-like growth factors expression [56,132]. Therefore, decreasing body adiposity and insulin resistance could contribute to reducing risks of cancer. Moreover, whole grains may also affect breast cancer through regulating hormone levels. Whole grain foods are sources of phytoestrogen-like isoflavones, which could influence hormone levels and activities [57]. Dietary fiber in whole grains may decrease circulating estrogen concentrations by suppressing bacterial β-glucuronidase activity, raising transient time and peristaltic activities in the gut, which inhibits the reabsorption of estrogens in the colon and increases the excretion of estrogens in feces [84]. Phytoestrogens and dietary fiber together would decrease inner estrogen concentrations, inhibit tumor development, and weaken early stage cancer risk marker expression. In addition, many bioactive phytochemicals are uniquely found in commonly consumed whole grains and are highly effective in modulating signaling pathways that are involved in breast cancer occurrence and progression. Thus, the consumption of whole grains containing these micronutrients may be beneficial for breast cancer prevention as hitherto discussed. The potential of whole grain consumption in reducing breast cancer risk is also supported by epidemiological and experimental evidences. 

However, several important issues remain to be solved. Firstly, current epidemiological studies on the relationship between whole grain consumption and breast cancer took “whole grains” as the consumption parameter, but did not set different grain species as individual parameters for assessment. Concentration of the bioactive phytochemicals vary significantly among different cereals and growing regions. Future studies should consider using standardized specific species and cultivars with unique bioactive compositions as an experimental factor. The dietary pattern of different geographic regions should also be taken into consideration. Secondly, while many comprehensive studies have evaluated the anti-breast cancer properties of grain extracts including wheat, sorghum, and rice, only a few have closely examined the specific phytochemicals and their mechanisms. Structure activity relationship characteristics of the phytochemicals and their pharmacophoric functionalities should be comprehensively studied. The inability to identify the individual active components is a great obstacle to interpreting the existing results’ correlation between whole grain consumption and breast cancer risk. Thirdly, the new molecular and genetic classification of cancer has provided alternative aspects for the diagnosis and treatment of breast cancer [133,134]. Focus should be placed on the various molecular targets of different breast cancer phenotypes by comprehensively studying the anticancer phytochemicals of whole grains. Moreover, chemo/radio-resistance remains to be one of the major obstacles during metastatic breast cancer treatment. Therefore, the combination of cereal phytochemicals with cytotoxic chemotherapy and/or selective BCSC-target agents could be a novel adjuvant therapy strategy for combating cancer metastasis. In addition, tumor immunotherapy has received increased attention due to its high specificity and low body toxicity [26]. The ability of cereal bioactive components to modulate immune response in breast cancer should be explored in future. More efforts should be placed on the in vivo studies and human intervention trials investigating the preventive effect of whole grains and/or their phytochemicals. Last, but not least, exploiting the synergistic potential of whole grain phytochemicals with chemotherapeutic and targeted therapies should be seriously and comprehensively considered. It is of paramount importance that future research focuses on integrated approaches that combine dietary or nutraceutical interventions with systemic endocrine therapies or chemotherapies. 

## 5. Conclusions

Breast cancer is one of the most pervasive and fatal carcinomas worldwide, which represents a serious threat to women’s health. There is an urgent need for the development and implementation of novel and effective therapeutic strategies that are affordable and accessible. Whole grains account for a large proportion of the dietary structure in developing countries around the world. Epidemiological studies have demonstrated that whole grains (and its products) are associated with reduced risk of breast cancer. Therefore, increasing whole grain consumption in daily dietary structure is a practical strategy for breast cancer prevention. 

Whole grains are rich in unique bioactive phytochemicals, which have been proven to be effective at targeting signaling pathways in breast cancer. Utilizing these phytochemicals synergistically with current standard therapy strategies may be a feasible approach for breast cancer treatment. Future studies on the health benefits of whole grains phytochemicals should consider targeting drug resistance and optimizing delivery method. 

## Figures and Tables

**Table 1:** Feature of breast cancer subtypes (modified from [19,20]).

Molecular Subtype	IHC Marker (ER/PR/HER2)	Frequency (%)	Proliferation Cluster	Gene Markers	Histologic Grade	Prognosis
Luminal A	ER+ PR+ HER-2-	50–60	Low	ESR1, GATA3, KRT8, XBP1, FOXA1, TFF3, CCND1, LIV1	Low	Excellent
Luminal B	ER+ PR+/- HER-2+	10–20	High	ESR1, GATA3, KRT8, XBP1, FOXA1, TFF3, SQLE, LAPTM4B	Intermediate/High	Intermediate/Bad
Basal-like	ER- PR-/+ HER-2-/+	10–20	High	KRT5, CDH3, ID4, FABP7, KRT17, LAMC2, TRIM29	High	Bad
HER2-enriched	ER-/+ PR-/+ HER-2+	10–15	High	ERBB2, GRB7	High	Bad
Normal breast-like	ER+/- PR+/- HER-2-	5–10	Low	VIM, MMP2/14, COL3A1, TIMP1, CD36, FABP4, ITGA7	Low	Intermediate
Claudin-low	ER- PR- HER-2-/+	12–14	High	CD24(-), CD44(+)	High	Bad

IHC: immunohistochemistry; ER: estrogen receptor; PR: progesterone receptor; +: positive; –: negative; +/-: predominantly positive; -/+: predominantly negative.

**Table 2:** The potential health benefits of bioactive phytochemicals extracted from whole grains.

Bioactive Phytochemicals	Major Sources	Potential Health Benefits	References
Alkylresorcinols	Wheat, rye	Cancer prevention; obesity reduction	[64,65]
Avenanthramide	Oat	Neutralizing free radicals, cancer prevention	[66,67]
**Phenolics**			
Anthocyanins	Barley, rice, sorghum	Neutralizing free radicals; inflammatory inhibition; cancer prevention	[40,45,68,69,70,71]
Lignans	Wheat, rye	Cancer prevention; hormone modulation; reducing the risk of cardiovascular disease	[72,73]
Flavones	Rye, barley, sorghum	Neutralizing free radicals; cancer prevention.	[45,68,69,74]
Tannins	Barley, sorghum	Improve urinary tract health; reducing risk of cardiovascular disease	[45,68]
**Carotenoids**			
α-carotene/β-carotene	Wheat, barley, millet	Neutralizing free radicals; reducing heart disease risks	[61,75]
**Phytosterols**			
Sterols	Wheat, barley, oat	Lowering blood cholesterol levels; reducing lipid accumulation; cancer prevention; reducing cardiovascular disease risks	[66,76,77]
Stanols	Wheat, maize, barley	Lowering blood cholesterol levels; reducing lipid accumulation; cancer prevention; reducing cardiovascular disease risks	[66,76,77]
**Non-starchy Polysaccharide**			
Insoluble dietary fiber	Wheat	Cancer prevention; lowering plasma cholesterol; reducing insulin resistance level	[75,78]
β-Glucans	Oat, barley	Reducing the risk of cardiovascular disease; lowering the level of low-density lipoprotein and total cholesterol, cancer prevention	[45,66,67,79]
**Tocols**			
Tocopherols	Barley, oat	Inhibiting lipid peroxidation; reducing the risk of cardiovascular disease; reducing stroke risks	[66,80]
Tocotrienols	Barley, oat	Inhibiting lipid peroxidation; reducing the risk of cardiovascular disease; reducing stroke risks	[66,80]

**Table 3:** Epidemiological and clinical evidences of breast cancer and whole grain intakes.

Natural Product (diet)	Study Type	Case/Participants	OR/RR (95%CI)	Conclusion	Reference
Whole grain	Meta-analysis of cohort and case-control studies	11,589/131,151 (4 cohort and 7 case-control studies)	Summary RR: 0.84 (0.74–0.96, *P*= 0.009, *I^2^* = 63.8%)	High intake of whole grains might be inversely associated with reduced breast cancer risks, but the inverse association was only observed in case-control not cohort studies.	[5]
Cereal dietary fiber	Meta-analysis of perspective studies	14,694/502,082 (six prospective studies)	Summary RR: 0.96 (0.90–1.02, *I^2^* = 5%)	Cereal dietary fibers have an inverse association with breast cancer risk.	[55]
Dietary fiber	Meta-analysis of perspective studies	16,848/712,195 (10 prospective cohort studies)	Summary RR: 0.89 (0.83–0.96, *I^2^* = 0%)	There was a significant inverse dose-response association between dietary fiber intake and breast cancer risk.	[84]
Whole grain bread	Case-controlled study	306/309	OR:0.61 (0.37–0.99)	Resistant starch containing foods (whole grain wheat bread) may reduce breast cancer risk.	[83]
Whole grains	Case-controlled study	250/250	OR:0.49 (0.29–0.82)	Whole grain consumption more than 7 times/week was associated with reduced risk of breast cancer.	[6]
Dietary Fiber	Prospective cohort study	2833/90534 (Follow-up: 20 years)	RR: 0.84(0.70–1.01; *Ptrend* = 0.04)	Higher fiber intakes during adolescence and early adulthood could reduce breast cancer risk.	[81]
Whole grain contained food	Prospective cohort study	3235/90516 (Follow-up: 22 years)	RR: 0.82(0.70–0.97; *Ptrend* = 0.03)	High whole grain food intake may be associated with lower breast cancer risk before menopause.	[82]
Whole and refined grain food	Prospective cohort study	124/3184 (Follow-up: 22 years)	HR: 0.53(0.33–0.86)	Higher consumption of whole grain food may protect against breast cancer, with 47% lower breast cancer risk.	[56]

OR: Odds ratios; RR: Relative Risks; HR: hazard ratios; CI: confidence intervals.

**Table 4:** Summary of health benefits of whole grains (and/or its components) on breast cancer.

Source	Constituents	Study Model (Cell Lines/Animal)	Mechanism	Reference
*in vitro*				
Wheat	Germinated wheat flour	Human breast cancer ER-positive MCF-7& TNBC MDA-MB-231	Up-regulation of apoptosis	[91]
Barley	Young barley and its methanolic extract	Human breast cancer MCF-7	Up-regulation of apoptosis, through lower metabolic activity, inhibition of proliferation, and cell cycle arrest in S phase	[92]
Foxtail millet	Total phenolic extracts	Human breast cancer MDA-MB-231	Proliferation inhibition	[61]
Synthetic analog of oat avenanthramide	Dihydroavenanthramide D	Human breast cancer MCF-7	Cancer cell invasion inhibition through the down regulation of MMP-9 activity and suppression of MAPK/NF-κB and MAPK/AP-1 pathway	[93]
Oat	Avenanthramide-C	Human breast cancer MDA-MB-231	Activation of apoptosis and caspases activity, positive annexin V staining and cell cycle arrest in sub G1 indicating DNA fragmentation	[94]
Wheat bran	Triticuside A	Human breast cancer MCF-7& MDA-MB-231	Activation of mitochondrial apoptosis pathway and Akt/mTOR signaling pathway, with downregulation of Mcl-1 and Bcl-2 and increase of cleavage of caspases-3, -7, -9, and PARP. Level of phospho-Akt and its downstream targets, mTOR, and P70 S6 kinase are also decreased	[95]
Rice bran	δ-Tocotrienol and Ferulic acid	Human breast cancer MCF-7	δ-tocotrienol and ferulic acid co-use synergistically inhibit cancer cell proliferation and induced cell arrest in the G1 phase	[96]
Rice bran	Arabinoxylan	Human breast cancer MCF-7; murine metastatic breast cancer 4T-1	Arabinoxylan increased the susceptibility of both types of cancer cells to paclitaxel by causing DNA damage, enhancing apoptosis, and inhibiting cell proliferation	[97]
Red rice bran	crude ethanolic extract of red rice bran	Human breast cancer MDA-MB-231	Decreased the secretion and activity of MMP-2 and MMP-9 reducing cells invasion	[98]
Rice bran	Glu-Gln-Arg-Pro-Arg	Human breast cancer MCF-7& MDA-MB-231	Anti-proliferation activity	[99]
Sorghum	Total sorghum extracts	Human breast cancer MCF-7, MDA-MB-231 & HER-2+/ER-SKBR-3	G1 phase arrest Down-regulation of the STAT5/IGF-1R and STAT3/VEGF pathway	[100]
Red sorghum bran	Anthocyanins	Human breast cancer MCF-7	Anti-proliferation activity	[101]
Red sorghum bran	3-Deoxyanthocyanins	Human breast cancer MCF-7	Anti-proliferation activity P53 gene up-regulation; bcl-2 gene down-regulation	[102]
*in vivo*				
Sorghum (Hwanggeumchal sorghum)	Total sorghum extracts	BALB/c nude mice	Breast cancer tumor suppression; down-regulation of STAT5b/IGF-1R and STAT3/VEGF signal pathways; breast-to-lung metastasis blockage	[100]
Black rice	Anthocyanins	BALB/c nude mice	Decreased activity of urokinase-type plasminogen activator (u-PA), and reduced transplanted tumor growth and inhibited pulmonary	[103]
Barley	Young barley	Sprague-Dawley female rats	Decrease in tumor incidence and average tumor volume; Caspase-3/caspase-7 increased; Ki67 decreased	[92]

## Abbreviations

BC	breast cancer
BCSC	breast cancer stem cells
BRCAs	breast cancer growth suppressor proteins
EMT	epithelial-mesenchymal transition
ER	estrogen receptor
ErbB2	erythroblastic leukemia viral oncogene homolog 2
FAK	focal adhesion kinase
Gal	galactose
Glc	glucose
HER-2	human epidermal growth factor receptor-2
IGF-1R	insulin-like growth factor 1 receptor
MMPs	matrix metallopeptidases
PR	progesterone receptor
RAF	rapidly accelerated fibrosarcoma
MEK	MAPK/Erk kinase
JNK	Jun N-terminal kinase
u-PA	urokinase-type plasminogen activator
SRC	steroid receptor coactivator
STAT	signal transducer and activator of transcription
VEGF	vascular endothelial growth factor
p130Cas	p130 Crk-associated substrate
